# Probing Immune-Mediated Clearance of Acute Middle Ear Infection in Mice

**DOI:** 10.3389/fcimb.2021.815627

**Published:** 2022-01-24

**Authors:** Kalyan K. Dewan, Colleen Sedney, Amanda D. Caulfield, Yang Su, Longhuan Ma, Uriel Blas-Machado, Eric T. Harvill

**Affiliations:** ^1^ Department of Infectious Diseases, College of Veterinary Medicine, University of Georgia, Athens, GA, United States; ^2^ Department of Pathology, College of Veterinary Medicine, University of Georgia, Athens, GA, United States

**Keywords:** otitis media, *Bordetella bronchiseptica*, natural infection, adaptive immunity, protective immunity

## Abstract

Acute otitis media (AOM) is commonly caused by bacterial pathobionts of the nasopharynx that ascend the Eustachian tube to cause disease in the middle ears. To model and study the various complexities of AOM, common human otopathogens are injected directly into the middle ear bullae of rodents or are delivered with viral co-infections which contribute to the access to the middle ears in complex and partially understood ways. Here, we present the novel observation that *Bordetella bronchiseptica*, a well-characterized respiratory commensal/pathogen of mice, also efficiently ascends their Eustachian tubes to colonize their middle ears, providing a flexible mouse model to study naturally occurring AOM. Mice lacking T and/or B cells failed to resolve infections, highlighting the cooperative role of both in clearing middle ear infection. Adoptively transferred antibodies provided complete protection to the lungs but only partially protected the middle ears, highlighting the differences between respiratory and otoimmunology. We present this as a novel experimental system that can capitalize on the strengths of the mouse model to dissect the molecular mechanisms involved in the generation and function of immunity within the middle ear.

## Introduction

It is estimated that bacterial infections of the middle ears, acute otitis media, affect 80% of the human population, usually at some point in their early lives (Bluestone and Doyle, 1988; Alsarraf et al., 1999; Monasta et al., 2012; Vos et al., 2016; Liese et al., 2014; DeAntonio et al., 2016; Tong et al., 2018). Yet despite its prevalence and a long history of human affliction (Goycoolea et al., 2019; Olivé-Busom et al., 2021), the disease has been difficult to study. A large number of these infections are caused by nasopharyngeal pathobionts that reach and colonize the middle ear *via* the Eustachian tube often during viral co-infections (Bluestone et al., 1992; Soriano, 1997; Marom et al., 2012). Clinical studies indicate that the propensity of infants to suffer repeated episodes of these infections is associated with compromised immunity (Urschel, 2010; Bardou et al., 2020; Pichichero, 2020), reflected in lower detected levels of cytokines, circulating antibody titers (Basha et al., 2014), and vaccine-induced memory B cells (Basha et al., 2014; Basha and Pichichero, 2015). However, efforts to gain a more mechanistic understanding of early interactions between otopathogens and host immunity face experimental challenges, underscored by the limited ability of human-specific otopathogen to efficiently ascend the Eustachian tubes of rodents that are commonly used as animal models to study otitis media (Piltcher et al., 2002; Melhus and Ryan, 2013; Park and Lee, 2013; Davidoss et al., 2018). To overcome this limitation, pathogens are injected directly into their middle ears to establish infections (Park and Lee, 2013). Despite the biological caveats and technical challenges, direct middle ear inoculations have been productively used to establish and study complex aspects of middle ear infections (Babl et al., 2002; Bouchet et al., 2003; Novotny et al., 2021) and remain the leading approach to gain insight into the innate immune mechanisms of host response and pathogen-specific aspects of middle ear infection (Schilder et al., 2016).

One consequence of direct inoculation of pathogens in the ears is that it bypasses the natural progression of infection that initiates from the nasopharynx, reaching the middle ear *via* the Eustachian tube, leaving gaps in our understanding of the complex interactions and host responses gradually unfolding in this context. In particular, it remains poorly understood how immune mechanisms are engaged and how they function in the prevention/resolution of naturally progressing infection, and how they might be subverted by pathogens to allow persistence. A complimentary experimental system that allows the study of the natural progression of middle ear infections and the corresponding host immune responses could substantially improve our understanding and guide efforts to prevent and cure them (Principi and Esposito, 2020).

We have previously described the ability of *Bordetella pseudohinzii* to naturally establish persistent infections in the middle ears of mice, apparently for life (Dewan et al., 2019). Here, we report that the closely related species, *Bordetella bronchiseptica*, a pathogen and persistent colonizer of the upper respiratory tract of many mammals (Parkhill et al., 2003), also naturally establishes robust infections in the middle ears of laboratory mice starting from low numbers of nasally inoculated bacteria. However, unlike *B. pseudohinzii*, which establishes persistent infections, *B. bronchiseptica* is gradually cleared from the middle ears of immunocompetent hosts, presenting an opportunity to study the immunological mechanisms involved in resolving middle ear infections. Using immunodeficient mouse strains and experimental approaches designed to probe immune mechanisms, we establish important contributions of both B- and T-cell functions to the clearance of middle ear infection. The data from this initial examination highlight the considerable advantages in the use of mouse-specific tools of immunology to experimentally investigate both the basic functioning of effective immunity in the middle ear as well as the complex interactions between bacterium and host immune response in the context of natural middle ear infections.

## Materials and Methods

### Bacterial Cultures and Preparation of the Inocula

*Bordetella bronchiseptica* RB50 was grown on Bordet–Gengou (BG) agar (Becton Dickson, Ref: 248200) with 10% sheep blood (HemoStat, Ref: 644000-1) and 20 µg/ml streptomycin (Acros, Organics) for 2 days at 37°C. Liquid cultures were obtained by growing the bacterium in Stainer–Scholte broth (Stainer and Scholte, 1970) at 37°C with shaking at 200 rpm (VWR, Model: Advanced 3500 Orbital Shaker) overnight. Serial dilutions were performed in PBS to obtain an estimated 500 colony-forming units (CFUs) in 5 μl PBS for the nasal, low dose–low volume inoculation and confirmed by plating.

### Mouse Experiments

Wild-type (C57BL/6/J), B-cell deficient [*Ighm* ^tm1Cgn^/J (alias: muMt^−^)], T-cell deficient [*Tcrb^tm1Mom^ Tcrd^tm1Mom^/J*], and *Rag-1^−/−^* (*Rag1^tm1Mom^/J*) female mice were obtained from Jackson Laboratories (Bar Harbor, USA) and were housed in a specific pathogen-free facility at The University of Georgia. Mice were inoculated by gently pipetting an estimated 500 CFUs of bacteria suspended in 5 µl PBS, onto the external nares of mice anesthetized with 5% isoflurane (Pivetal, Ref: P151A).

To test for colonization of organs, mice were sacrificed by CO_2_ inhalation (1.5 L/min) for removal of organs. Lungs and trachea were collected via ventral dissection of the the thoracic cavity. For nasal cavities: the dorsal bones, internasal septum and soft tissue of lateral and ventral surfaces enclosing the nasal cavities was collected; middle ears were collected by removal of the ventral jaw to expose the left and right bullae which were then carefully excised. Harvested samples were collected in 1 ml of sterile PBS containing ceramic beads (2.8mm, Omni International) and homogenized using a bead homogenizer (Fischerbrand, Model: Bead Mill 24). Serial dilutions were plated on Bordet–Gengou agar to quantify CFUs. The bacterial colonies were identified by their characteristic pale white color, dome-shaped colony morphology, and typical zone of β-hemolytic clearance on blood agar plates. For the time course of colonization, mice were euthanized at the indicated days post-inoculation (dpi).

### Adoptive Transfer Experiments

Immune serum for adoptive transfer was prepared from blood collected by cardiac puncture from convalescent mice (56 dpi) inoculated with a high dose of *B. bronchiseptica* delivered to reach the lungs by inhalation (5 × 10^5^ CFUs delivered in 50 μl of PBS). A total of 200 μl of serum was injected intraperitoneally 2 h before mice were challenged with a low dose (500 CFUs) of *B. bronchiseptica* delivered intranasally in 5 μl PBS by inhalation. Single-cell suspensions of splenocytes for adoptive transfer were prepared from spleens collected in RPMI media 1640 (Gibco) from either naive or convalescent (56 dpi) mice inoculated with 5 × 10^5^ CFUs (delivered in 50 μl PBS) of intranasally delivered *B. bronchiseptica*. Single-cell suspensions of splenocytes were prepared by passing the organ through a 70-μM mesh and pipetting several times. Finally, cells were resuspended in 200 μl of PBS to be intraperitoneally injected into recipient *Rag-1^−/−^* mice.

### Histopathology

Forty-eight, 5-week-old, female C57BL/J6 mice were divided into two groups and inoculated intranasally with either 5 μl of PBS or containing 500 CFUs of *B. bronchiseptica.* Mice were evaluated by histopathology on 3, 7, 14, and 28 dpi. Following fixation in neutral-buffered, 10% formalin solution and subsequent decalcification in Kristensen’s solution, transverse sections were made through the middle and inner ear. Tissues were subsequently processed, embedded in paraffin, sectioned at approximately 5 mm, and stained with hematoxylin and eosin. Histopathological examination consisted of evaluation of the ear for the incidence (presence or absence), severity, and distribution of inflammation.

### ELISA Assay

Assays were performed according to established laboratory protocols. Briefly, 96-well Nunc microtiter plates (ThermoScientific, Ref: 80040LE 0910) were coated with heat-killed *B. bronchiseptica* and incubated in a humidified chamber at 37°C for 4 h. Following binding, the plate was then blocked with PBS with 0.1% Tween 20 and 1% BSA overnight at 4°C. Four microliters of serum was used to estimate total IgG using two-fold serial dilutions to titrate out end points using goat–anti-mouse HRP-conjugated antibodies (Invitrogen). Reactions were developed with SureBlue™ (Sera Care, Ref: 5120 0076) and terminated with 1 M HCl. Color intensities were determined at an OD of 450 nm and arbitrary titer values were determined to be the reciprocal of the lowest dilution in which an OD of 0.1 was obtained.

### Statistical Analysis

Data generated were statistically evaluated by Student’s *t*-test and two-way ANOVA using the statistical analyses package of GraphPad Prism (V2.0).

### Study Approval

All animal experiments were carried out in strict accordance with the recommendations in the Guide for the Care and Use of Laboratory Animals of the National Institutes of Health. The protocol was approved by the Institutional Animal Care and Use Committee at the University of Georgia, Athens, GA, United States (“Bordetella-Host interaction”: A2016 02-010-Y3-A9).

## Results

### A Natural Example of Efficient Middle Ear Infection in Mice

**Figure 1:**
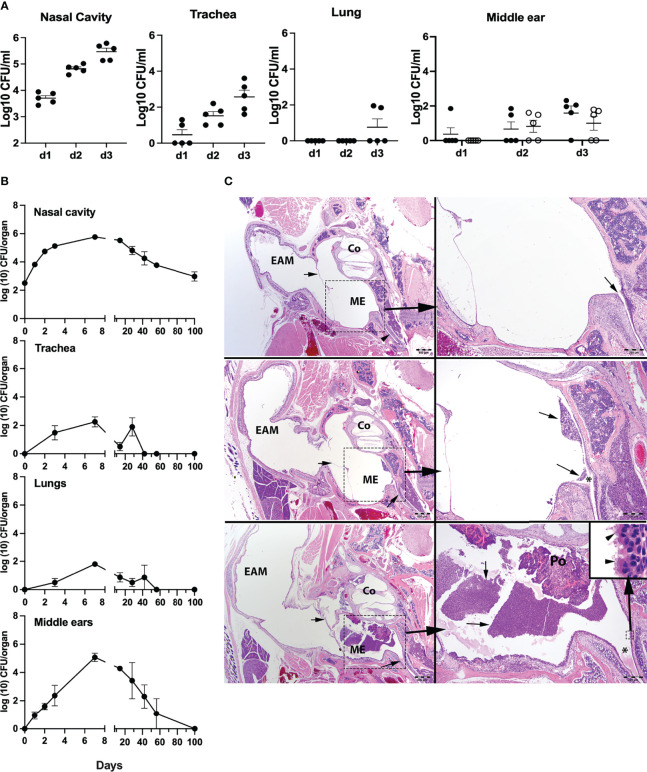
Colonization and growth of *Bordetella bronchiseptica* in the respiratory tract and middle ears of C57BL/6J mice. **(A)** Graphs represent the *B*. *bronchiseptica* colony-forming units (CFUs) recovered (log_10_/ml) from the nasal cavity, trachea, lungs, and middle ears (left to right) from individual C57BL6J mice (filled circles, *n* = 5 per group). For the middle ears, circles represent bacteria recovered from the left (filled circle) and right (open circle) middle ear bulla of an individual mouse. **(B)** Graphs represent the number of CFUs recovered over time from the nasal cavity, trachea, lungs, and middle ears of groups of C57BL/6J mice (*n* = 4 per group). Filled circles represent the mean value ± SEM. **(C)** Images represent developing inflammation in the middle ears from C57BL/6J mice. HE stains. *Top panel*: uninfected. In the left image, the small arrows point to the tympanic membrane, and in the lower right, to the Eustachian tube as it enters the middle ear. Scale bar = 500 μm. The right-side image represents higher magnification of the dashed boxed region of the middle ear. Scale bar = 200 μm. *Middle panel*: 3 dpi with *B*. *bronchiseptica*. The left image, the small arrows point to minimal accumulation of inflammatory exudate within the middle ear. Scale bar = 500 μm. The right-side image represents higher magnification of the dashed boxed region of the middle ear. Arrows point to minimal accumulation of neutrophils and macrophages arranged about or near the Eustachian tube (*) as it enters the middle ear. Scale bar = 200 μm. *Lower panel*: 7 dpi with *B*. *bronchiseptica*. In the left image, the arrowhead points to a moderate accumulation of inflammatory exudate within the middle ear. Scale bar = 500 μm. The right-side image represents higher magnification of the dashed boxed region of the middle ear. Arrows point to a densely cellular collection of neutrophils and macrophages arranged about or near the Eustachian tube (*) as it enters the middle ear, just below an ear polyp (Po; incidental finding). Scale bar = 200 μm. Inset (small dashed box): Bacteria attached to the cilia (arrowheads) on the epithelia lining the middle ear by the entrance of the Eustachian tube. Scale bar = 10 μm. EAM, external auditory meatus; Co, cochlea; ME, middle ear in bony tympanic bulla.

We had earlier reported that the mouse respiratory pathogen *B. pseudohinzii* persistently colonizes the respiratory tract and middle ears of mice (Ivanov et al., 2016; Dewan et al., 2019). *Bordetella bronchiseptica*, a broad host-range respiratory pathogen of significant veterinary interest (Parkhill et al., 2003; Mattoo and Cherry, 2005), has also been noted to persist indefinitely in the nasal cavities of laboratory mice, but has not been described as colonizing the middle ear. To examine whether the ability to colonize the middle ear is specific to *B. pseudohinzii*, or is shared with *B. bronchiseptica*, we inoculated groups of C57BL/6J mice (*n* = 5) with an estimated inoculum of 500 CFUs of *B. bronchiseptica*, delivered in a 5-μl droplet of PBS to the external nares. The early colonization profile of the respiratory tract (nasal cavity, trachea, and lungs) and middle ears was then examined over the course of 3 days (**Figure 1A**). *Bordetella bronchiseptica* colonized and grew efficiently to roughly 0.5 million CFUs in the nasal cavities and also reached and grew to lower numbers in the trachea (~2.75 × 10^2^ CFUs) of all mice 3 dpi. Only two mice showed evidence of being colonized in the lungs at 3 dpi. Importantly, we observed that four of the five mice had either one or both their middle ears colonized by hundreds of bacteria by day 3 indicating that the pathogen naturally, efficiently, and rapidly colonizes this organ. To confirm this observation and examine the long-term colonization profile of the pathogen in the middle ears, we inoculated groups of C57BL/6J mice (*n* = 4) and examined bacterial colonization loads for days 1, 2 (for the nasal cavity and middle ears), 3, 7, 14, 28, 42, 56, and 100 (for the nasal cavity, trachea, lungs, and middle ears) (**Figure 1B**). *Bordetella bronchiseptica* colonized and grew efficiently in the nasal cavities of all mice, reaching ~5 × 10^5^ CFUs a week after inoculation, then gradually declining to about 10^3^ CFUs, but persisting stably in all mice thereafter. However, the pathogen spread poorly to the trachea and lungs, reaching only a few hundred CFUs at day 3 and was not detected by day 56. As seen earlier, colonization of the middle ears began within 24 h following inoculation and bacterial numbers continued to grow to reach a peak of about 10^5^ CFUs at 7 dpi. However, by 14 dpi, these bacterial numbers had substantially decreased and continued to decline thereafter, approaching the limit of detection by 56 dpi and were fully cleared from both middle ears of all animals by 100 dpi.

Histopathological analysis of hematoxylin–eosin-stained sections of the middle ears on days 3 and 7 p.i. (peak bacterial load) revealed the presence of *B. bronchiseptica* in the middle ears with a range of severity of inflammation noted, including the moderately inflamed sections shown (**Figure 1C** and **Supplementary Table 1**). While not all mice showed detectable inflammation, these findings indicate that *B. bronchiseptica* can efficiently reach and grow in numbers within each of the middle ears of the mice and can induce significant inflammation within the middle ears. However, the host is capable of suppressing the continued expansion of the pathogen, and ultimately clears the infection from this organ. Importantly, once infection had been cleared, both middle ears remain free of the pathogen indicating the attainment of bilateral protective immunity, despite the persistent bacterial colonization of the nasal cavity.

### Clearance of *Bordetella bronchiseptica* From the Middle Ears Is Dependent on Adaptive Host Immunity

**Figure 2:**
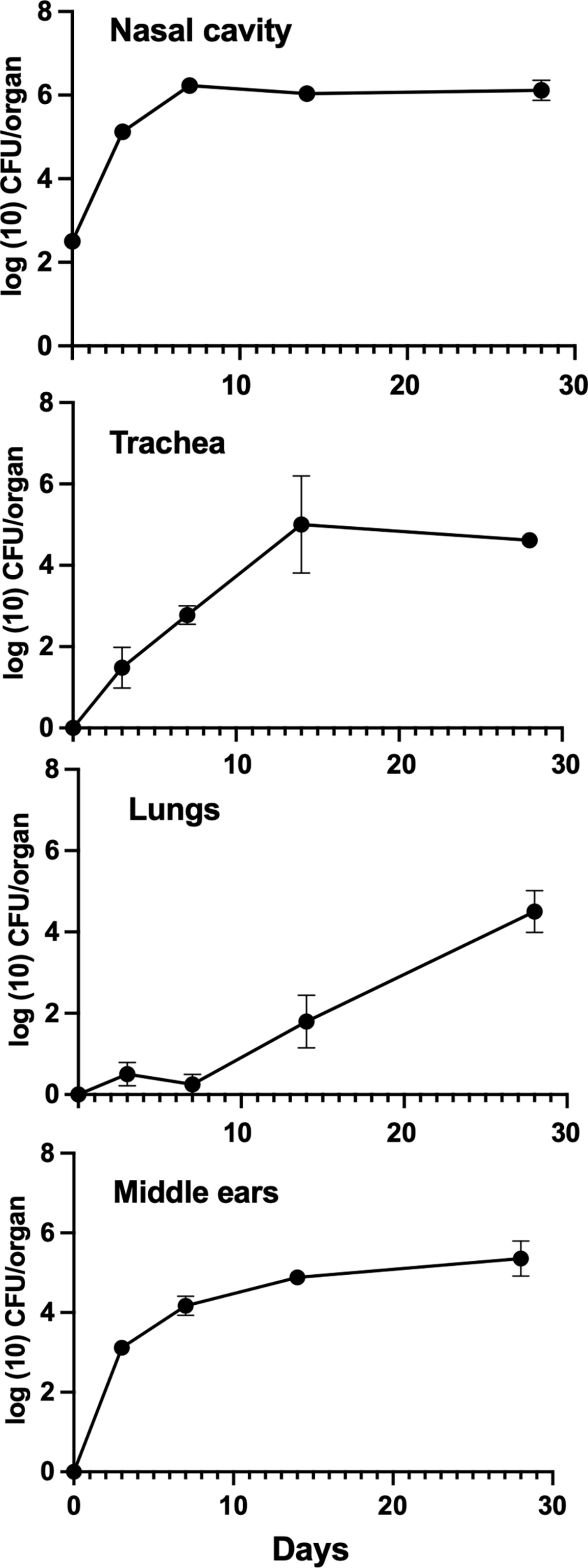
Growth of *B*. *bronchiseptica* in *Rag-1^−/−^* mice. Graphs represent the number of *B*. *bronchiseptica* RB50 CFU recovered from the nasal cavity, trachea, lungs, and middle ears of groups of *Rag-1^−/−^* mice over time following inoculation. Error bars represent the standard deviation (*n* = 4).

The gradual clearance observed in C57BL/6J mice suggested that adaptive immune mechanisms are involved in protecting the middle ears from *B. bronchiseptica*. To test the roles of T and B cells in this, we examined the persistence of *B. bronchiseptica* in the middle ears of *Rag-1^tm1Mom^* (*Rag-1^−/−^*) mice, which lack mature T and B cells. Groups of *Rag-1^−/−^* mice were inoculated with *B. bronchiseptica* as above and the numbers determined in the respiratory tract and middle ears thereafter (**Figure 2**). *Bordetella bronchisept*ica colonized and initially grew in the nasal cavities of *Rag-1^−/−^* mice similar to the wild type. However, after reaching peak levels of nearly a million CFUs at 7 dpi, the numbers did not decrease but persisted at this peak level.

*Bordetella bronchiseptica* also colonized the tracheas and lungs of *Rag-1^−/−^* mice, but unlike in wild-type mice, the numbers of bacteria gradually increased in the lower respiratory tract reaching ~10^4^ CFUs by 28 dpi, indicating that T and B cells play a significant role in resisting the spread to and/or growth within the lower respiratory tract. *Bordetella bronchiseptica* also efficiently colonized the middle ears of *Rag-1^−/−^* mice and grew to similar numbers as the wild type by day 3. Unlike in wild-type mice, where bacterial numbers began to decrease within 2 weeks, the number of CFUs recovered from the middle ears continued to increase, clearly showing that mature B and T cells are critical for controlling and clearing middle ear infections. Bacterial numbers continued to rise beyond day 28 until animals became ill and had to be euthanized (data not shown).

### Both T and B Cells Are Required for Controlling Middle Ear Infections

**Figure 3:**
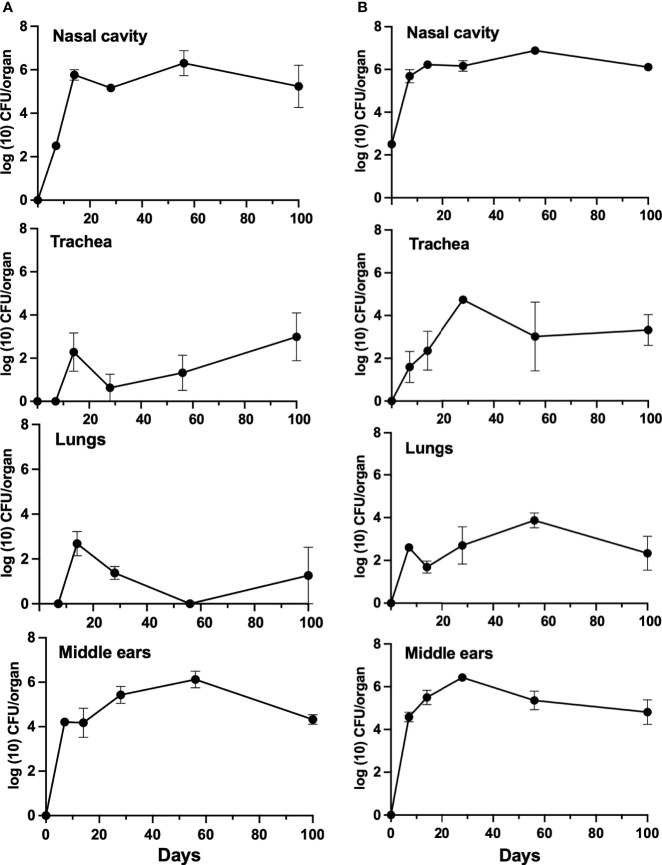
Growth of *B*. *bronchiseptica* in B- and T-cell-deficient mice. Graphs represent the number of CFU recovered from the nasal cavity, trachea, lungs, and middle ears of groups of B-cell-deficient (left, panel **A**) and T-cell-deficient (right, panel **B**) mice. Error bars represent the standard deviation (*n* = 4).

To examine the individual B- and T-cell contributions to the clearance of *B. bronchiseptica*, mice deficient in either B cells or T cells were inoculated with *B. bronchiseptica* as above and bacterial growth and persistence examined over the course of 100 days (**Figure 3**). *Bordetella bronchiseptica* efficiently colonized and grew to similarly high numbers (~10^6^ CFUs) in the nasal cavities of both B-and T-cell-deficient mice by 7 dpi and persisted till the end of the experiment (100 dpi). T-cell-deficient mice had higher numbers of *B. bronchiseptica* in the trachea and lungs at most time points, and B-cell-deficient mice appeared to sporadically clear the infection from the lower respiratory tract. However, within the middle ears, *B. bronchiseptica* grew rapidly to over 10^4^ CFUs by 3 dpi in both strains of mice. The numbers continued to grow thereafter, plateauing around 10^6^ CFU. In summary, T and B cells are of different importance in the lower respiratory tract, but both are required for the control and clearance of *B. bronchiseptica* from the nose and from the middle ear, highlighting differences in immune function in respiratory organs and the middle ear.

### Adoptively Transferred Antibodies Partially Protect Middle Ears

Given the important role of B cells in controlling the bacterial load in the middle ear and the contribution of T cells to the efficient generation of an antibody response, we examined the ability of circulating IgG antibodies to protect the middle ears from *B. bronchiseptica*. We had earlier reported (Kirimanjeswara et al., 2003) that adoptively transferred serum antibodies from convalescent mice effectively protected the lower respiratory tract from *B. bronchiseptica* but had little if any effect in the nasal cavity. To examine what protection antibodies could confer to the middle ears, convalescent serum obtained from high-dose *B. bronchiseptica*-infected mice (56 dpi), which generates high titers of anti-*B. bronchiseptica* IgG, was adoptively transferred into groups of *Rag-1^−/−^* mice (*n* = 4). Two hours later, the mice were challenged with 5 × 10^5^ CFUs of *B. bronchiseptica* delivered in 50 μl PBS, depositing bacteria in high numbers throughout the respiratory tract. As controls, we included a group of mice that did not receive the antibodies. We then examined the colonization load on 3 dpi.

**Figure 4:**
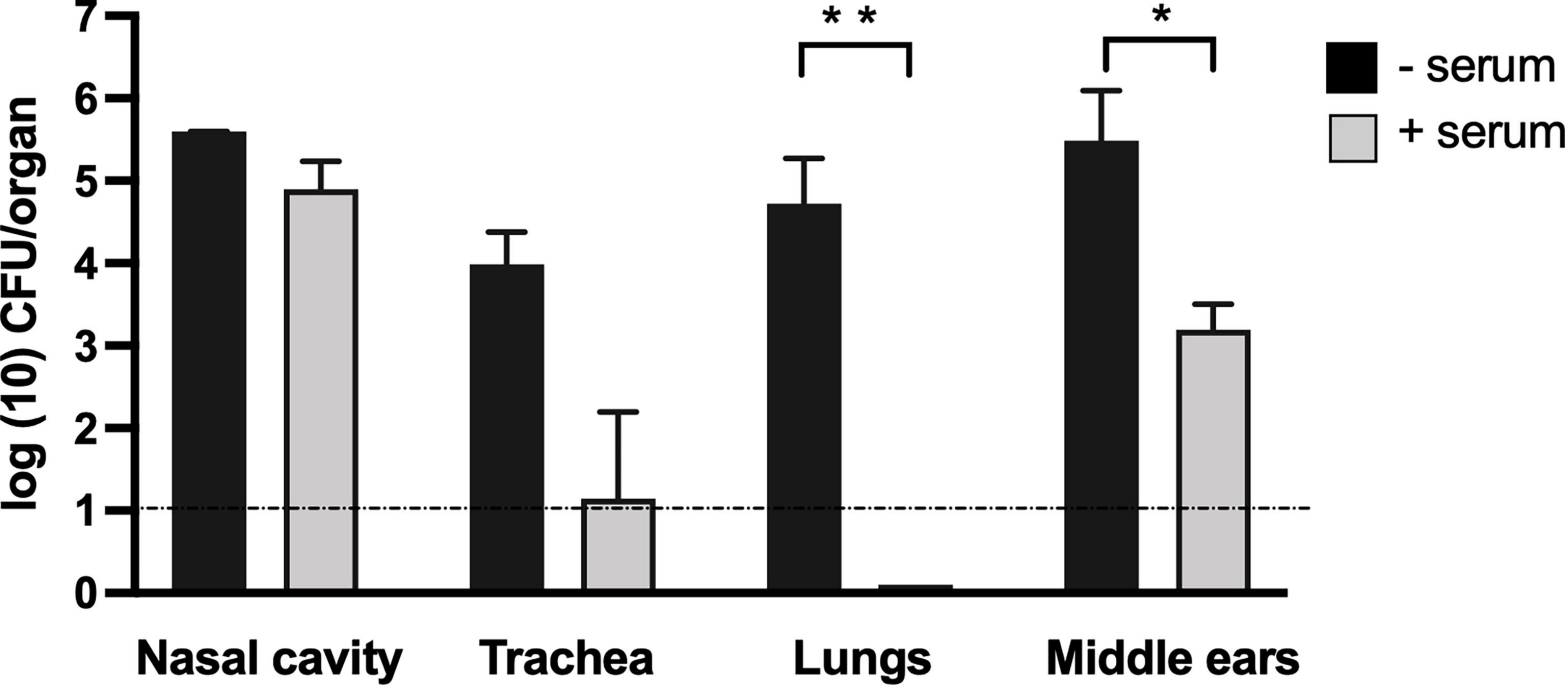
Adoptively transferred convalescent serum protects the lower respiratory tract but is less effective in the nasal cavity and middle ears. Graphs represent the number of CFUs recovered on 3 dpi from the nasal cavity (NC), trachea (TR), lungs (LNG), and middle ears (ME) of groups of C57Bl/6J mice. The adoptively transferred convalescent serum was collected from C57BL/6J mice (light gray columns) or control mice that received no serum (black columns). Dashed line represents the limit of detection. Error bars represent the standard deviation (*n* = 4). *p < 0.05, ** <0.001.

As shown in **Figure 4**, approximately 10^5^ CFUs were recovered from the nasal cavities of both antibody-treated and untreated groups of mice, indicating that serum antibodies had little if any impact in the nose. In contrast, adoptively transferred antibodies nearly and completely cleared *B. bronchiseptica* from the trachea and lungs, respectively. These results are similar to those described by Kirimanjeswara et al. (2003), demonstrating different effects of serum antibodies in the upper and lower respiratory tracts. Interestingly, adoptively transferred antibodies substantially reduced, but did not clear, bacterial numbers in the middle ears. These data indicate that serum antibodies can have a substantial but incomplete impact on *B. bronchiseptica* colonization of the middle ear, and that impact is measurably different from the effects in the upper and the lower respiratory tracts.

### Splenocytes Effectively Protect the Lungs and Partially Protects the Nasal Cavity and Middle Ear

**Figure 5:**
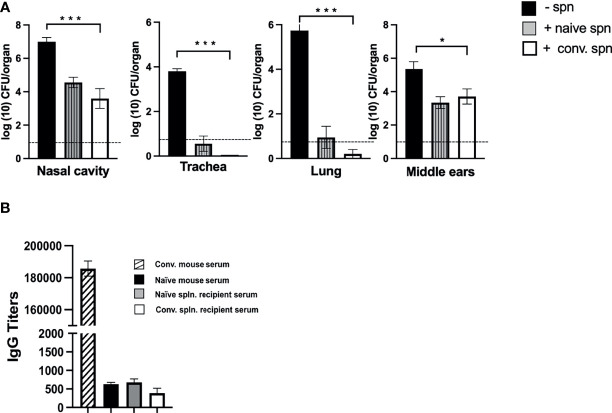
Effect of splenocyte transfer on the growth of *B*. *bronchiseptica* in *Rag-1^−/−^* mice. **(A)** Graphs represent the number of CFUs of *B*. *bronchiseptica* recovered at day 28 post-inoculation from the respiratory tract and middle ears of *Rag-1^−/−^* mice that either received no splenocytes (black) or mice that received splenocytes from naive mice (dark gray striped column) or splenocytes from convalescent mice (white column) 2 h before being inoculated with 500 CFUs of bacteria (*n* = 4 mice per group). Error bars represent the standard deviation. Dotted line represents the limit of detection. **(B)** Graphs represent the anti-*B. bronchiseptica* serum IgG titers (arbitrary units) from mice studied in **(A)** [control serum from d56 p.i. convalescent mice (striped column); no splenocytes (black); splenocytes from naive mice (dark gray), convalescent splenocytes (white)]. Error bars: ± SEM; *p < 0.05, *** <0.0001.

As circulating serum antibodies showed limited impact on preventing colonization of the middle ears, we proceeded to examine whether adoptively transferred immune cells of the peripheral lymphoid system were capable of clearing *B. bronchiseptica* from the middle ears. One spleen equivalent of splenocytes from either naive or convalescent (56 dpi) C57BL/6J mice were intraperitoneally transferred into groups of *Rag-1^−/−^* mice. Two hours after this transfer, the mice were inoculated with 500 CFUs (5 μL PBS) of *B. bronchiseptica* along with an untreated group of mice as a control. After 28 days post-inoculation, the bacterial burden in the respiratory tract and middle ears of the different groups was examined (**Figure 5A**).

While the untreated control group of *Rag-1^−/−^* mice showed the expected high bacterial burden in the respiratory tract and middle ears, no bacteria could be detected in the tracheas and lungs of immune splenocyte-treated mice and only a few bacteria, close to the limits of detection, were recovered from some mice treated with naive splenocyte (**Figure 5A**). Importantly, for both these groups of treated mice, we observed reduced numbers of bacteria in the nasal cavity and middle ears, indicating that the transferred splenocytes controlled the unrestricted growth of bacteria otherwise observed in *Rag-1^−/−^* mice and reduced their numbers by ~99% to the levels seen for the wild-type C57BL/6J mice at the corresponding day 28 time point of infection (refer to **Figure 1**). We considered whether this reduction in bacterial load might be antibody mediated, but ELISA assays for total IgG in the sera of these mice showed no significant difference in titers compared with PBS-treated C57BL/6J or untreated *Rag-1^−/−^* mice (**Figure 5B**).

These observations suggest that middle ear protective immunity is directed *via* T-cell-mediated immune mechanisms, likely involving tissue-resident T cells. We did observe a noticeably higher percentage of T-cell populations with resident memory phenotype (CD69^+^, CD103^+^) in the middle ears at day 100 compared with uninfected controls (**Supplementary Figure 1**). Although this difference was not statistically significant in this experiment, the possibility arises that these could be contributing to clearance and/or subsequent protection from reinfection of the middle ears which is the focus of ongoing investigations.

## Discussion

Clinical studies have associated repeated episodes of middle ear infections with compromised immune status. This is reflected in lower detected levels of cytokines, circulating antibody titers, and memory-specific B cells against vaccine. However, obtaining a more direct mechanistic understanding of the host–pathogen interactions of middle ear infections has been problematic with the lack of models that provide a means to study the progression of natural disease—i.e., initial colonization, infection, and disease resolution.

*Bordetella bronchiseptica*, a naturally adapted respiratory tract pathogen in many mammals (Dewan and Harvill, 2019) that is well studied in the respiratory tracts of laboratory mice, also infects their middle ears. When delivered in relatively small numbers in a small volume of PBS that restricts initial colonization to the nasal cavity, infections of the middle ears develop rapidly and consistently, allowing for the study of the natural course of disease progression. We observed that *B. bronchiseptica* rapidly colonizes the middle ears and grows, reaching hundreds of thousands of CFUs in a week: evidence that the bacterium possesses the factors necessary to overcome the physiological and innate immune barriers of the host. What is particularly noteworthy is that despite the bacteria in the nasal cavities persisting at high levels, their numbers in the middle ears begin decreasing after the first week, reaching the limits of detection by 7–8 weeks. These profoundly different outcomes, despite following somewhat similar kinetics of early infection in the nasal cavities and middle ears, suggest qualitative differences in the immune mechanisms between the two associated organs in the upper respiratory tract. Interestingly, the low inoculation dose we employed, while sufficient to launch robust infections in these organs, fails to consistently reach the lower respiratory tract, corroborating the observations that *B. bronchiseptica* is primarily an upper respiratory tract pathogen. In addition, the low inoculation doses used do not generate the high titers of circulating IgG antibodies normally observed when high numbers of the bacteria are inoculated into the lungs, highlighting the potential caveats with using unnaturally large inocula. Associations between low antibody titers in middle ear fluid and acute otitis media were noted decades ago (Sloyer et al., 1976). Our observations that middle ear infections are cleared, despite very low serum IgG antibody titers, suggested that serum IgG antibodies play a modest role in clearing *B. bronchiseptica* from the organ. This fact is further supported by our observation that adoptively transferred convalescent serum, with high titers of *B. bronchiseptica*-specific IgG, cleared >99.9% of the bacteria from the lungs, but had little effect in protecting the nasal cavities and only partially protected the middle ears. However, the observation that muMT^−^ (B-cell-deficient) mice were unable to clear *B. bronchiseptica* from the middle ear suggests that secretory IgA plays an important role. This agrees with the reports of IgA secreting cells being the dominant antibody-producing cells detected by ELISPOT in the middle ear mucosal cells (Suenaga et al., 2001) and with the model of IgA forming B cells being primed in the NALT and migrating to the middle ears to differentiate into plasma cell (Kodama et al., 2000; Kaur et al., 2012).

Interestingly, once *B. bronchiseptica* is cleared from the middle ears, the organ remains protected from subsequent reinfection by bacteria that persist in the nasal cavities, indicative of the acquisition of a middle ear-specific immunity, which, once established, protects the organ from reinfection. T-cell-deficient mice failed to clear middle ear infections indicating a critical role for T cells. Our experiments on the protective effect of adoptively transferred splenocytes in *Rag-1^−/−^* mice support the hypothesis that subsets of peripheral lymphoid cells are migrating to the upper respiratory tract and middle ears to establish a population of memory cells. We did note a marginal increase in populations with CD4^+^ resident memory cell phenotype (CD69^+^, CD103^+^) that arose at later points (100 dpi) in the middle ears of C57BL/6J mice which correlated with the reduction in bacterial loads there (**Supplementary Figure 1**). These may have a role in providing protection from reinfection of the middle ears; however, more detailed investigations on the migration of T cells to the middle ears and of the pathogen specificity of this arising population would need to be investigated. Notwithstanding the increases in resident memory cell populations, the mechanisms involved in the reduction of bacterial loads from the middle ears and those that prevent the organ from being recolonized are unknown and it is the current focus of our investigations.

Although *B. bronchiseptica* is not a primary human pathogen, the efficient colonization of the middle ears of mice and the unparalleled tools available to study the immune response in mice are likely to make this experimental system valuable for the study of the mechanistic detail of the workings of functional immunity in the middle ear. In addition, *B. bronchiseptica* harbors an array of well-studied virulence factors, for which the corresponding deleted mutant strains are available to probe host–pathogen interactions (Cotter et al., 1998; Mattoo et al., 2000; Mattoo et al., 2001; Irie et al., 2004; Dewan et al., 2017; Gestal et al., 2019; Ma et al., 2021). Identifying the factors involved in overcoming physiological barriers to ascend the host Eustachian tube and/or overcome innate host immunity to naturally colonize the middle ears would significantly inform our understanding of how middle ear infections are established. Importantly, as exemplified by the preliminary experiments we conducted here, the mouse model supported with the comprehensive immunological/immunogenetic resources available allows highly incisive questions on features of host immunity that are either induced or suppressed during the control/clearance of middle ear infections.

Finally, it is important to consider the caveats to both the study of human pathogens in non-human hosts they poorly infect and the study of non-human pathogens in their natural hosts (Lairmore and Khanna, 2014). The strengths and weaknesses of each should be acknowledged and carefully weighed against the research question being asked. It may be necessary to embrace the truism attributed to George Box that “All models are wrong. Some models are useful” (Box and Draper, 1987). All models are by default approximations; whether they provide useful information that can inform our understanding and guide our development of improved treatments and preventatives for human disease remains the question.

## Data Availability Statement

The raw data supporting the conclusions of this article will be made available by the authors, without undue reservation.

## Ethics Statement

The animal study was reviewed and approved by the Institutional Animal Care and Use Committee, University of Georgia.

## Author Contributions

KD and EH conceived the study. KD and EH designed the experiments. KD, CS, AC, YS, LM, and UB-M performed the experiments. KD, CS, AC, YS, LM, UB-M, and EH analyzed the data. KD and EH wrote the manuscript. All authors contributed to the article and approved the submitted version.

## Funding

This work was supported by grants AI149787, DC018496, AI156293, and AI159347 from the National Institutes of Health to EH. The funders had no role in the study design, data collection and interpretation, or the decision to submit the work for publication.

## Conflict of Interest

The authors declare that the research was conducted in the absence of any commercial or financial relationships that could be construed as a potential conflict of interest.

## Publisher’s Note

All claims expressed in this article are solely those of the authors and do not necessarily represent those of their affiliated organizations, or those of the publisher, the editors and the reviewers. Any product that may be evaluated in this article, or claim that may be made by its manufacturer, is not guaranteed or endorsed by the publisher.
