# Supplementary material for: Probing Immune-Mediated Clearance of Acute Middle Ear Infection in Mice
Source: Front Cell Infect Microbiol. 2022 Jan 24;11:815627. doi: 10.3389/fcimb.2021.815627 (PMC8818953; doi:10.3389/fcimb.2021.815627)
Supplement: Supplementary file 1 [file DataSheet_1.pdf]

Supplementary Material

			Inflammation	
DPI	Animal ID	Group	Sev	Dist
3	1	Infected	2	1
	2	Infected	2	1
	3	Infected	0	0
	4	Infected	0	0
7	1	Infected	0	0
	2	Infected	0	0
	3	Infected	0	0
	4	Infected	2	1
	5	Infected	0	0
	6	Infected	1	1
	7	Infected	3	2
	8	Infected	0	0
28	1	Naïve	0	0
	2	Naïve	0	0
	3	Naïve	0	0
	4	Naïve	0	0
	5	Infected	1	1
	6	Infected	2	1
	7	Infected	0	0
	8	Infected	0	0
	9	Infected	0	0
56	1	Infected	2	1
	2	Infected	0	0
	3	Infected	2	1
	4	Infected	2	1
	5	Infected	0	0

Supplementary Table 1. Table shows the number of mice examined and found with inflammatory lesions in the middle ears on days 3, 7, 28 and 56 following low dose intranasal inoculation with *B. bronchiseptica* (500 CFU in 5 μL of PBS). Not all mice showed evidence of inflammation and where detected (highlighted rows) the severity of inflammation (Sev) ranged from mild to moderate. Histopathological scores were assigned as Grades 0 (No significant histopathological alterations); 1 (Minimal); 2 (Mild); 3: (Moderate); 4 (Severe). Distribution scoring Key: 1- Focal; 2-Multifocal or Bilateral; 3-Diffuse.

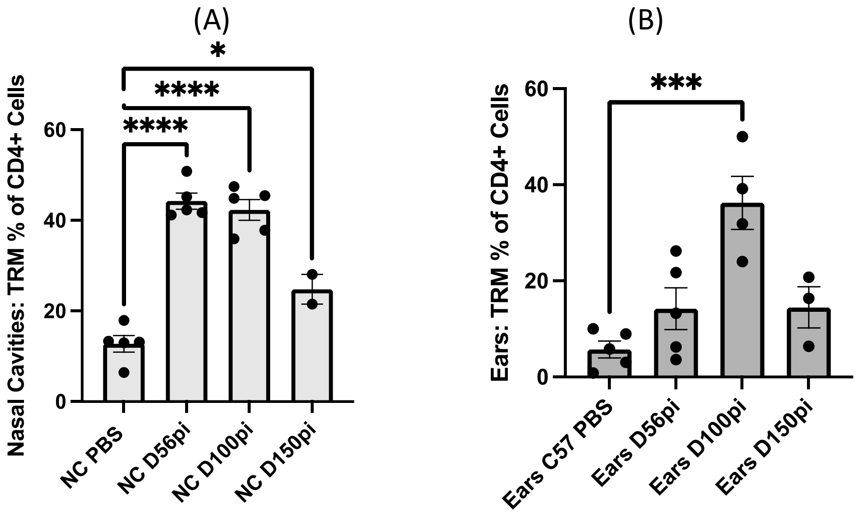

Supplementary Figure 1.

Percentage of CD4+ T-cells expressing Tissue resident memory cell markers ($CD69^+$, $CD103^+$) in the nasal cavity and middle ears

Mice (n=5) were euthanized by CO_2 inhalation. Nasal cavity (A) and middle ear bullae (B) were harvested, placed in 1mL of RPMI 1640 medium and homogenized via a syringe plunger against a 40µm cell strainer. Cell suspensions were centrifuged at 1,500rpm for 10 minutes and remaining red blood cells were lysed with ACK lysing buffer. After washing with PBS, the cells were incubated with 1µl Zombie aqua (Biolegend) for 20 minutes, washed again, and incubated with 1µl Fc Block (Biolegend) for 30 minutes. Surface marker staining was added to each sample from a master mix of antibodies. Cells were fixed, washed, and resuspended in 250µl FACS buffer. Flow cytometry (Acea Novocyte Quanteon) was performed to sort T cells and T cell subsets. The data were analyzed with FlowJo 10.0.
